# Guided domino lithography for uniform fabrication of single-digit-nanometer scale plasmonic nanoantenna

**DOI:** 10.1515/nanoph-2022-0694

**Published:** 2023-03-28

**Authors:** Dong Kyo Oh, Yeseul Kim, Jaekyung Kim, Inki Kim, Junsuk Rho

**Affiliations:** Department of Mechanical Engineering, Pohang University of Science and Technology (POSTECH), Pohang 37673, Republic of Korea; Department of Biophysics, Institute of Quantum Biophysics, Sungkyunkwan University, Suwon 16419, Republic of Korea; Department of Intelligent Precision Healthcare Convergence, Sungkyunkwan University, Suwon 16419, Republic of Korea; Department of Chemical Engineering, Pohang University of Science and Technology (POSTECH), Pohang 37673, Republic of Korea; POSCO-POSTECH-RIST Convergence Research Center for Flat Optics and Metaphotonics, Pohang 37673, Republic of Korea; National Institute of Nanomaterials Technology (NINT), Pohang 37673, Republic of Korea

**Keywords:** cascade domino lithography, extreme near-field enhancement, guided domino lithography, sub-nm resolution, ultra-sharp tip

## Abstract

Single-digit-nanometer scale plasmonic nanoantenna platforms are widely used in optical sensors, quantum plasmonics, and other applications. Uniform and reliable fabrications with a single-digit-nanometer resolution are desirable for diverse quantum nanophotonic device applications, but improving the process yield and uniformity of the shape of the nanoantenna over the entire fabrication area remains a challenge. Here we report the guided domino lithography fabrication method for uniform ultra-sharp nanoantenna arrays. We use a collapsing of unstable photoresist nanostructures with a guide structure to uniformly fabricate ultra-sharp bowtie photoresist masks. We directly compare the yields of the conventional and the guided domino lithography under the optimized electron beam exposing and development conditions. Furthermore, we conduct a rigorous analysis to verify the electric field enhancement effect from ultra-sharp bowtie nanoantennas fabricated with different geometry. We believe that guided domino lithography can be a promising solution toward a practical manufacturing method for single-digit-nanometer plasmonic nanoantennas.

## 1 Introduction

When the excitation light matches the resonance condition of metallic nanoparticles or structures, free electrons on the surface are strongly coupled with the incoming electromagnetic field. The acceleration of electrons creates dipole oscillations and strongly confined and localized optical fields on the near and surface of metal nanoparticles, called localized surface plasmon resonances (LSPR) [1]. Plasmonic nanoantennas using LSPR have strong interaction with incoming optical fields, allowing unprecedented confinement of them such as quantum plasmonics [2–5] and enhanced light–matter interactions [6–8]. Various types of nanoantennas such as single metal nanosphere/nanorods/nanodisk [9–12], bowtie nanoantennas [13–16], and Yagi-Uda nanoantennas [17, 18] have been researched both numerically and experimentally so far. Most of all, the bowtie nanoantennas with a sharp tip nanostructure can effectively confine light with a little spectral shift in comparison with other types of nanoantennas, and the intensity of near optical fields can be amplified by millions of times when the light is incident [19].

To fabricate bowtie nanoantennas, electron beam lithography (EBL) is normally used [20–23]. The general steps to fabricate bowtie nanoantennas, called the lift-off process, are as follows. After the electron beam exposure on photoresists and the development process, metals are deposited on the substrate by an evaporation process. Then final bowtie structures can be formed by removing the residual resist. Since the EBL process uses an electron beam, it can make patterns with high resolution under 5 nm [24–26]. However, it is difficult to make ultra-sharp bowtie nanoantennas due to an electron beam proximity effect [27, 28]. Such proximity effect not only limits the patterning resolution of 10–20 nm but also causes large nanogaps and radius of curvatures (RoC) of exposed areas. Focused ion beam (FIB) milling can be another candidate to fabricate sharp bowtie nanoantennas. When accelerated ions are focused on the surface of metals, a strong collision of ions physically separates the remaining metal particles, excepting a bowtie-shaped area [29]. Since the FIB process doesn’t use any sensitive photoresist, nanoantennas can be formed directly on arbitrary surfaces [30]. However, the shape of bowtie nanoantennas fabricated by the FIB process is not vertical but tapered because the colliding ions are much bigger than electrons and the optical properties of nanoantennas differ from the designed one due to ion implantation [31, 32].

To improve the resolution of the EBL and FIB processes, capillary-force-induced collapsing processes have been introduced by applying mechanical deformation of fabricated photoresist structures in three dimensions [33–36]. They mainly use collapsed nanostructures to form very small nanocavities. One step further, capillary-force-induced collapse lithography (CCL) and cascade domino lithography (CDL) have been proposed to fabricate ultra-sharp bowtie nanoantennas with both a sub-nanometer scale sharpness and a single-digit-nanometer gap feature. CCL uses the cohesion and collapse of nanostructures to fabricate ultra-sharp nanostructures [37]. While the cohesion of nanostructures occurs when the capillary force is large enough to modify nanostructures, the collapse of nanostructures is highly dependent on the geometry of nanostructures. By optimizing the cohesion and collapse directions of nanostructures, ultra-sharp bowtie nanostructures with a sub-10 nm gap can be fabricated. On the other hand, CDL can fabricate ultra-sharp bowtie structures, using unstable photoresist structures [38]. The unstable nanostructures are formed due to e-beam resists with different development rates in the same developer. Ultra-sharp bowtie nanoantennas can be fabricated by mechanically falling these unstable island structures down and depositing metals. Despite these superior capacities to fabricate ultra-sharp bowtie nanoantennas, the formation area and uniformity of bowtie nanoantennas are still limited.

Here, we analyze collapsing parameters of unstable nanostructures and demonstrate guided domino lithography (GDL) to fabricate uniform nanoantenna arrays with single-digit-nanometer gaps in one area. we systematically investigate collapsing parameters of GDL such as a geometry of unstable photoresist structures and doses for uniform fabrication of ultra-sharp bowtie nanoantenna arrays. The geometry of nanostructures affects the collapsing directions of unstable nanostructures, which results in the uniform formation of ultra-sharp bowtie nanoantennas. E-beam doses also decide the successful collapsing area of unstable nanostructures, so rather excessive e-beam doses remove photoresist nanostructures. Thus, a proper e-beam dose should be controlled for the uniform formation of ultra-sharp bowtie nanoantennas. By using GDL, bowtie nanoantennas with various central degrees are fabricated and analyzed by rigorous finite element method (FEM) to confirm the distribution of enhanced near electric fields according to bowtie central degrees. The GDL to uniformly fabricate ultra-sharp bowtie nanoantennas with the enhancement of near electric fields has a potential to be applied to various newly developed but low-efficient optical signals such as photoluminescence, optical tweezers, surface enhanced Raman spectroscopy, and various plasmonic resonances.

## 2 Experimental section

### 2.1 Fabrication

**Figure 1:**
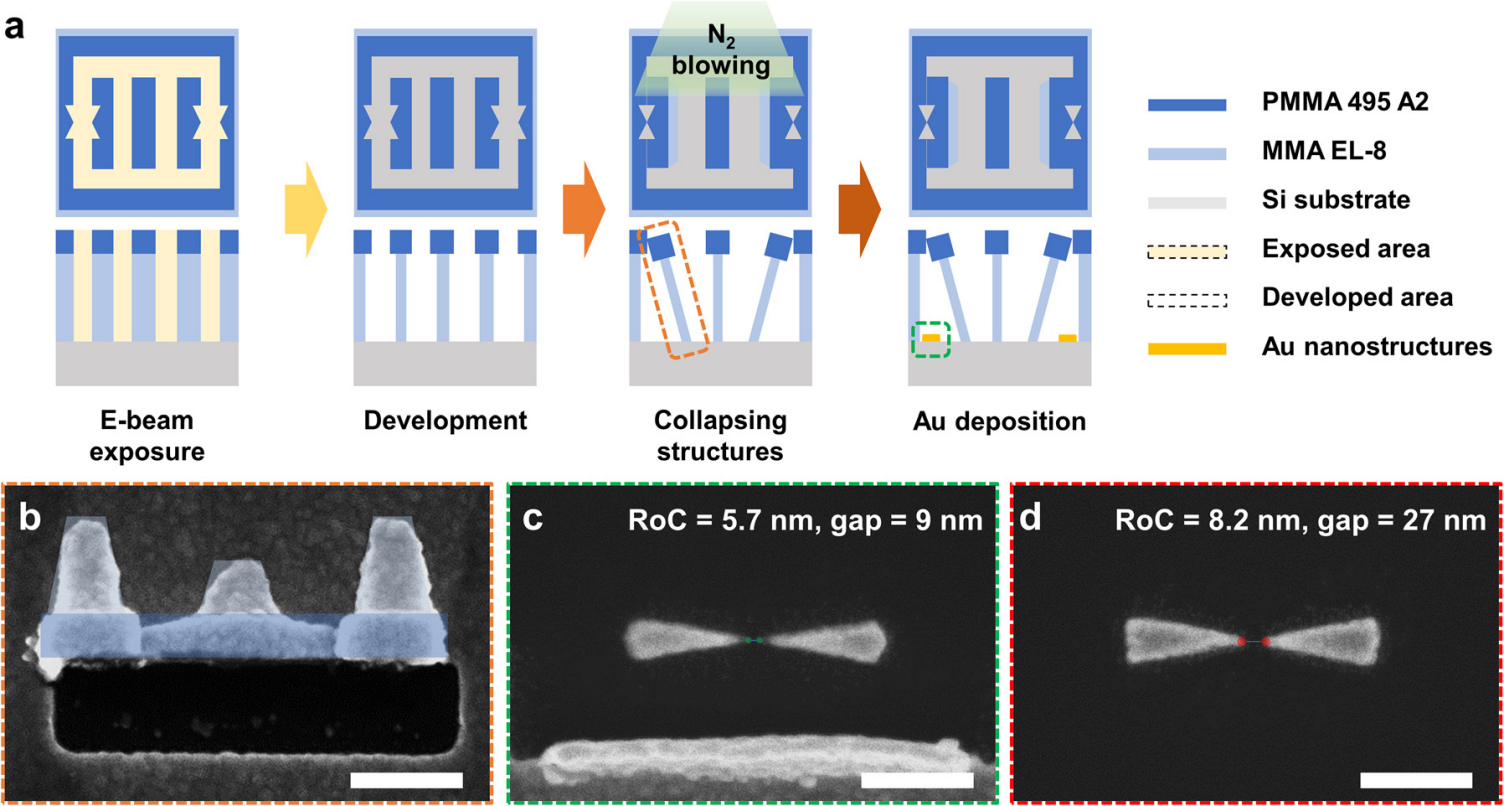
Fabrication of ultra-sharp bowtie nanoantenna using the mechanical collapse of nanostructures. (a) Schematics of the procedures of the guided domino lithography (GDL) process. Using different development rates of PMMA/MMA bilayers, unstable T-shaped nanostructures are collapsed by N_2_ blowing, following Au deposition for the fabrication of ultra-sharp bowtie structures. (b) SEM image of collapsed PMMA/MMA bilayer after the Au deposition and the lift-off process. The blue area and light blue area are developed PMMA 495 A2 and MMA EL-8 areas, respectively. The rest is a deposited Au residue area not covered by photoresist masks. (c) SEM image of ultra-sharp bowtie nanoantenna by GDL process. The radius of curvature (RoC) is about 5.7 nm and the gap size is about 9 nm. (d) SEM image of bowtie nanoantenna by EBL process. RoC is about 8.2 nm and the gap size is about 27 nm, which is larger than it is by the GDL process. All scale bars: 200 nm.

The overall process to fabricate ultra-sharp Au bowtie nanoantennas is shown in Figure 1a. First, the MMA (Microchem, MMA (8.5) MAA EL-8) layer was spin-coated (5000 rpm, 60 s) and baked for 5 min at 150 °C on the hotplate to acquire the film with a thickness of about 250 nm. Likewise, the PMMA (Microchem, 495 PMMA A2) layer was spin-coated (2000 rpm, 60 s) on the preformed MMA layer and baked for 5 min at 180 °C to acquire the film with a thickness of about 60 nm. Using an EBL process (Elionix ELS-7800, 100 kV, 100 pA), the bowtie nanoantenna area was defined on the MMA/PMMA bilayer. After the EBL process to different doses for about 2 min at each array, the MMA/PMMA bilayer resist was developed in MIBK:IPA 1:3 solutions for 15 min at 4 °C. Because MMA and PMMA have different solubility in the same developer, the development process results in unstable T-shaped nanostructures as shown in Figure 1b, and such a profile is favorable for the clean lift-off process because it can reduce sidewall deposition. A cold development with a longer development time enables gradual development in the MMA area affected by secondary electrons while the development in the PMMA layer is completed. The developed samples were rinsed with IPA for 30 s soon after. N_2_ was perpendicularly blown on the patterned area for 30 s to exclude unidirectional falling of the unstable nanostructures by directional N_2_ blowing. This resulted in the bidirectional collapse of the unstable T-shaped nanostructures, which form bowtie-shaped deposition masks by meeting the adjacent wall. On the mask, Cr (3 nm) and Au (60 nm) were deposited by an electron beam evaporator (KVT KVE-ENS4004), followed by the standard lift-off process. Because the angular spread of evaporated vapor flux can distort the sharpness, a vertical configuration between the vapor flux and the substrate was maintained. Finally, ultra-sharp Au bowtie nanoantennas were fabricated after the lift-off process, as shown in Figure 1c. For the general Au bowtie nanoantennas by the EBL process, the single PMMA layer was spin-coated (2000 rpm, 60 s) on the Si substrate and baked for 5 min at 180 °C to obtain a thickness of about 60 nm. After the EBL process, the exposed PMMA area was developed in MIBK:IPA 1:3 solutions for 15 min at 4 °C and were rinsed with IPA for 30 s. Following electron beam deposition of Cr (3 nm) and Au (60 nm) and the lift-off process, the general Au bowtie nanoantennas were fabricated, as shown in Figure 1d. The scanning electron microscopy (SEM) images were taken using a JEOL JSM-6700F field-emission SEM (typical operation voltage: 5 kV).

### 2.2 Numerical modeling details

We have chosen | ***E*** |^2^ as the field enhancement factor which is a proportional value of electromagnetic field intensity

(1) $$Enhancement{\left|E\right|}^{2}=\frac{{\left|E\right|}^{2}}{{\left|E_{0}\right|}^{2}}$$

where |*E*_0_| is the incident electric field amplitude, and ***E*** is the electric field of a specific point. The electromagnetic field distributions were evaluated using the commercial finite element method software COMSOL Multiphysics. To model the Au bowtie nanoantenna on the silicon substrate, the physical domain is surrounded by the perfectly matched layer (Figure S1). The scattered field calculation was performed at plane wave excitation. The optical properties of Au were taken from Johnson and Christy [39] and these of silicon were taken from Aspnes and Studna [40]. The mesh sizes were chosen to be smaller than the feature size depending on the structures. The parameter of Au bowtie and the bowtie geometry sweep process is shown and explained in Figures S2 and S3 which represent that sweep range and enhancement |*E*|^2^ results depending on the central angle of the bowtie (*θ*), gap distance (*g*), and radius of curvature (RoC) when the length of the bowtie (*L*) is 200 nm, and the height of the bowtie (*H*) is 60 nm.

## 3 Results and discussion

**Figure 2:**
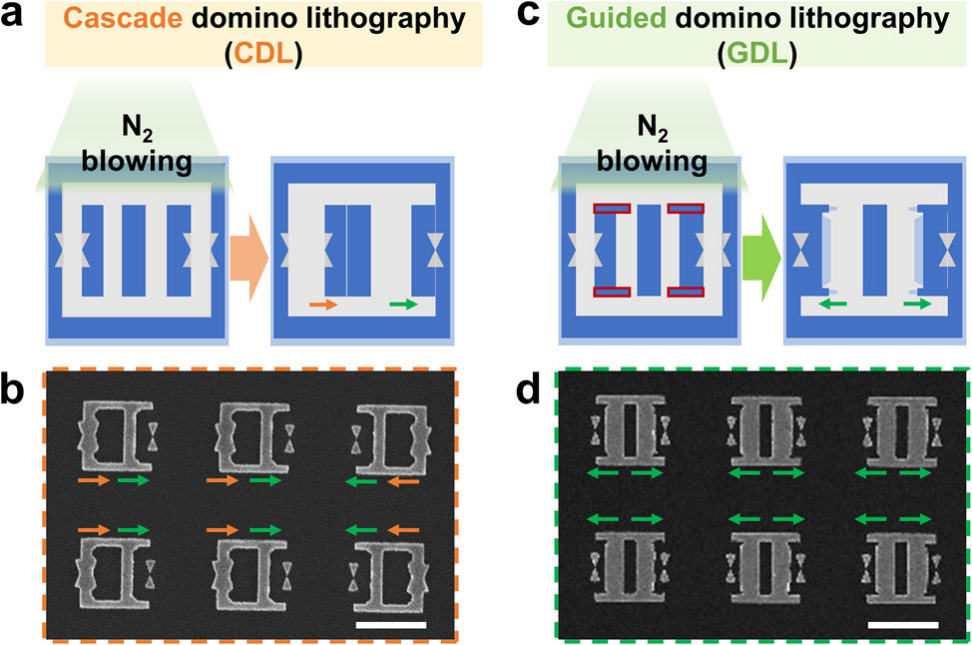
Comparison of CDL and GDL processes. (a) Schematic of the CDL process resulting in a random collapse of the unstable T-shaped island. (b) SEM image of randomly collapsed unstable nanostructures after the Au deposition and lift-off process. Due to uncontrolled falling directions of T-shaped nanostructures, 6 bowtie nanoantennas were fabricated in the 6 CDL arrays. (c) Schematic of the GDL process resulting in the directional collapse of the T-shaped nanostructures. (d) SEM image of bidirectional collapsing of the unstable nanostructures after the Au deposition. Due to the existence of guides to control the falling directions of nanostructures, 12 bowtie nanoantennas were fabricated in the 6 GDL arrays. All scale bars: 1 µm.

Both CDL and GDL processes use the mechanical collapse of unstable nanostructures, but they have slightly different collapsing nanostructures. The PMMA/MMA bilayers with different development rates have unstable T-shaped nanostructures after the development process. The perpendicular N_2_ blowing step on the developed area causes to collapse of the unstable T-shaped nanostructures. In the N_2_ blowing process, T-shaped nanostructures without guided structures fabricated by the CDL process are randomly collapsed, as shown in Figure 2a. This also affects fabrication rates of ultra-sharp bowtie nanoantennas in a constant area. Figure 2b indicates an SEM image of fabricated ultra-sharp bowtie nanoantennas in 6 CDL arrays. In one CDL array, only one bowtie nanoantenna is fabricated by the random collapse of the T-shaped nanostructures. On the contrary, the GDL process added with guided nanostructures can precisely control the collapsing directions of the unstable nanostructures, making more bowtie nanoantennas in the same area than the CDL process. Figure 2c shows the schematic of the GDL process. When the N_2_ is blown to the T-shaped nanostructures with guides, the collapsing is controlled to move in the opposite direction to the guide of the nanostructures. This controlled collapsing results in the uniform formation of two ultra-sharp bowtie nanoantennas in one GDL array, which is a doubled number of them by CDL, as shown in Figure 2d.

**Figure 3:**
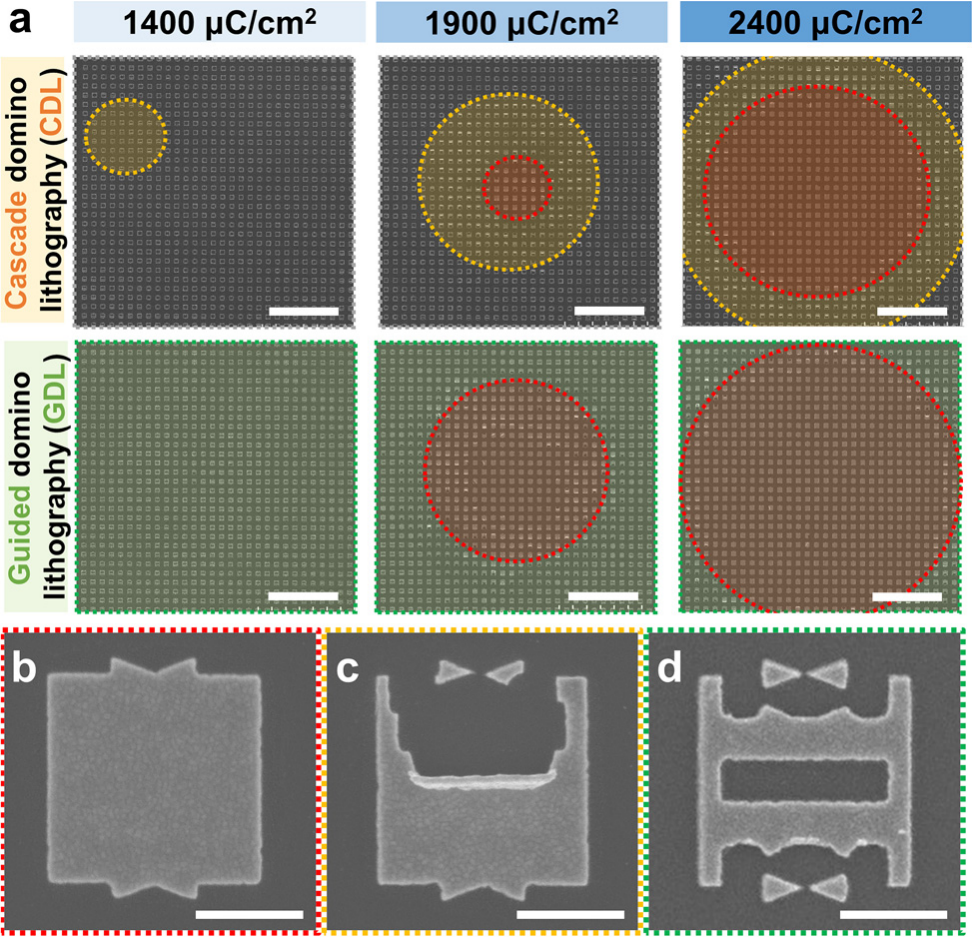
SEM images of ultra-sharp bowtie nanoantennas exposed by various exposing doses in CDL and GDL arrays. The development time was 15 min and exposing doses were varied: 1400, 1900, and 2400 µC/cm^2^, respectively. The green area is the region with the uniform fabrication of ultra-sharp bowtie nanoantennas, the yellow area is the region with occasionally misaligned bowtie nanoantennas, and the red area is the region without bowtie nanoantennas due to the absence of collapsed structures by excessive exposing doses. Scale bars: 1 µm. Corresponding SEM images of colored areas of (a): (b) red, (c) yellow, and (d) green. Scale bars: 500 nm.

Exposing dose is another crucial factor for the uniform fabrication of ultra-sharp bowtie nanoantennas. Figure 3a demonstrates areas of ultra-sharp bowtie nanoantennas by different exposing doses of the CDL and GDL processes, respectively. When the development proceeds for 15 min to the CDL sample exposed to 1400 µC/cm^2^, the collapse of the unstable T-shaped nanostructures is generated in a random position. By increasing the exposing dose to 1900 µC/cm^2^, the bowtie nanoantennas are formed in the center of the array. However, over-exposure also occurred, removing whole T-shaped nanostructures from the center area, as shown in Figure 3b. Despite the partial formation of bowtie nanostructures by a random collapsing of unstable nanostructures fabricated by the CDL process, a misalignment of collapsing nanostructures occurs as well, as shown in Figure 3c. When increasing the exposing dose to 2400 µC/cm^2^_,_ most nanostructures are over-exposed so they don’t exist after the development process. On the other hand, the guiding structures in the GDL process increase the fabrication rate of ultra-sharp bowtie nanostructures in the same exposing dose as the CDL process. Compared to the same exposing dose and development time of the CDL process, the GDL process can fabricate ultra-sharp bowtie nanoantennas in the whole area of GDL arrays at a development time of 15 min and exposure dose of 1400 µC/cm^2^, as shown in Figure 3d. This uniform formation of nanostructures is due to both the increased exposing area by the guides of T-shaped nanostructures and the easier directional collapsing of guided nanostructures. In the same exposing dose, the total exposed area of the unit nanostructure is increased in the GDL process, resulting in more unstable T-shaped nanostructures during the development process. In addition, the directional collapsing of unstable nanostructures with guides is much easier even with a little N_2_ blowing process. With light N_2_ blowing, lots of developed unstable nanostructures in the GDL arrays are not swept but collapsed, helping to fabricate more bowtie nanoantennas. Despite the uniform fabrication of ultra-sharp bowtie nanoantennas by the GDL process, excessive exposing dose over 1900 µC/cm^2^ causes unstable T-shaped nanostructures to disappear during the development process. The ultra-sharp bowtie nanoantenna formation by CDL and GDL processes at a large development time is also described in Figure S4.

**Figure 4:**
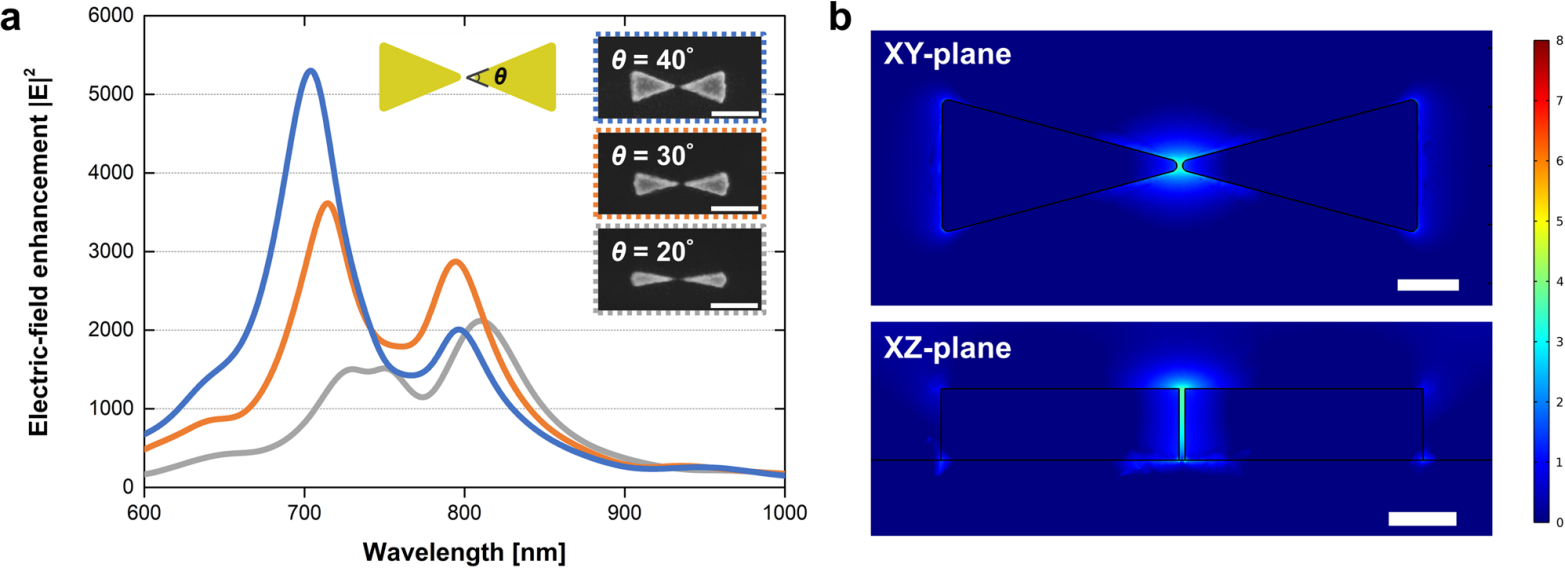
The electric-field enhancement and field distribution of Au bowtie nanoantenna. (a) Analysis of near-field enhancement | ***E*** |^2^ of 200 nm-length, 60 nm-height, 5 nm-gap Au bowtie nanoantenna with various central degrees and SEM images of Au bowtie nanoantennas with corresponding central degrees fabricated by the GDL process. Scale bars: 200 nm. (b) The distribution results of | ***E*** |^2^ field in log scale at 800 nm in XY and XZ planes. Scale bars: 50 nm.

As one verification of near-field enhancement in sharper and more uniform bowtie nanoantennas, we analyze the electric near-field enhancement of the designed ultra-sharp bowtie nanoantenna. Figure 4a shows the enhancement | ***E*** |^2^ at the wavelength from 600 nm to 1000 nm and SEM images of bowtie nanoantennas with corresponding geometries fabricated by the GDL process. Since the wavelength of maximum enhancement | ***E*** |^2^ can be modulated by changing the geometry, these bowtie structures can be applied from visible to a near-infrared range of light–matter interactions. Therefore, if there is an emitter, the resonance wavelengths of the bowtie nanoantennas can be matched with excitation and emission wavelengths. Figure 4b shows the distribution results of the | ***E*** |^2^ field in the log scale. The *XY* plane at *Z* = 60 nm and the *XZ* plane at *Y* = 0 show that the most enhanced point is near the bowtie center. The polarization-dependent field distribution results are added in Figure S5 and the polarization-dependent field distribution results are added in Figure S6.

## 4 Conclusions

We have demonstrated the GDL process to fabricate uniform ultra-sharp nanoantenna arrays by controlling both geometries of photoresist patterns and exposing doses. By adding guides to unstable T-shaped nanostructures, we can collapse them with the lower exposing dose and a little N_2_ blowing, which minimized the dissipation of over-developed structures during the development process. As a result, more ultra-sharp bowtie nanoantenna arrays have been fabricated by the GDL process. To secure the extreme near-field enhancement of fabricated ultra-sharp bowtie nanoantennas, an analysis of electric near-field enhancement in the nanogap between bowtie nanoantennas has been conducted. We believe that the uniform fabrication of ultra-sharp bowtie nanoantennas with single-digit-nanometer scale gaps by the GDL process has the potential to extremely amplify optical signals which are too weak to be applied such as photoluminescence, optical tweezers, surface enhanced Raman spectroscopy, and various plasmonic resonances.

## Supplementary Material

Supplementary Material Details
