# Supplementary material for: Guided domino lithography for uniform fabrication of single-digit-nanometer scale plasmonic nanoantenna
Source: Nanophotonics. 2023 Mar 28;12(8):1435–41. doi: 10.1515/nanoph-2022-0694 (PMC11501949; doi:10.1515/nanoph-2022-0694)
Supplement: Supplementary file 1 — Supplementary Material Details [file j_nanoph-2022-0694_suppl_001.docx]

Supplementary Material

**Guided domino lithography for uniform fabrication of single-digit-nanometer scale plasmonic nanoantenna platform**

Dong Kyo Oh^1,†^, Yeseul Kim^1,†^, Jaekyung Kim^1^, Inki Kim^2,3,*^, Junsuk Rho^1,4,5,6,*^

^1^ Department of Mechanical Engineering, Pohang University of Science and Technology (POSTECH), Pohang 37673, Republic of Korea

^2^ Department of Biophysics, Institute of Quantum Biophysics, Sungkyunkwan University, Suwon 16419, Republic of Korea

^3^ Department of Intelligent Precision Healthcare Convergence, Sungkyunkwan University, Suwon 16419, Republic of Korea

^4^ Department of Chemical Engineering, Pohang University of Science and Technology (POSTECH), Pohang 37673, Republic of Korea

^5^ POSCO-POSTECH-RIST Convergence Research Center for Flat Optics and Metaphotonics, Pohang 37673, Republic of Korea

^6^ National Institute of Nanomaterials Technology (NINT), Pohang 37673, Republic of Korea

^†^ These authors contributed equally to this work.

*Corresponding authors:

Dr. Inki Kim, inki.kim@skku.edu, Tel. +82-31-299-4794

Dr. Junsuk Rho, jsrho@postech.ac.kr, Tel. +82-54-279-2187

**
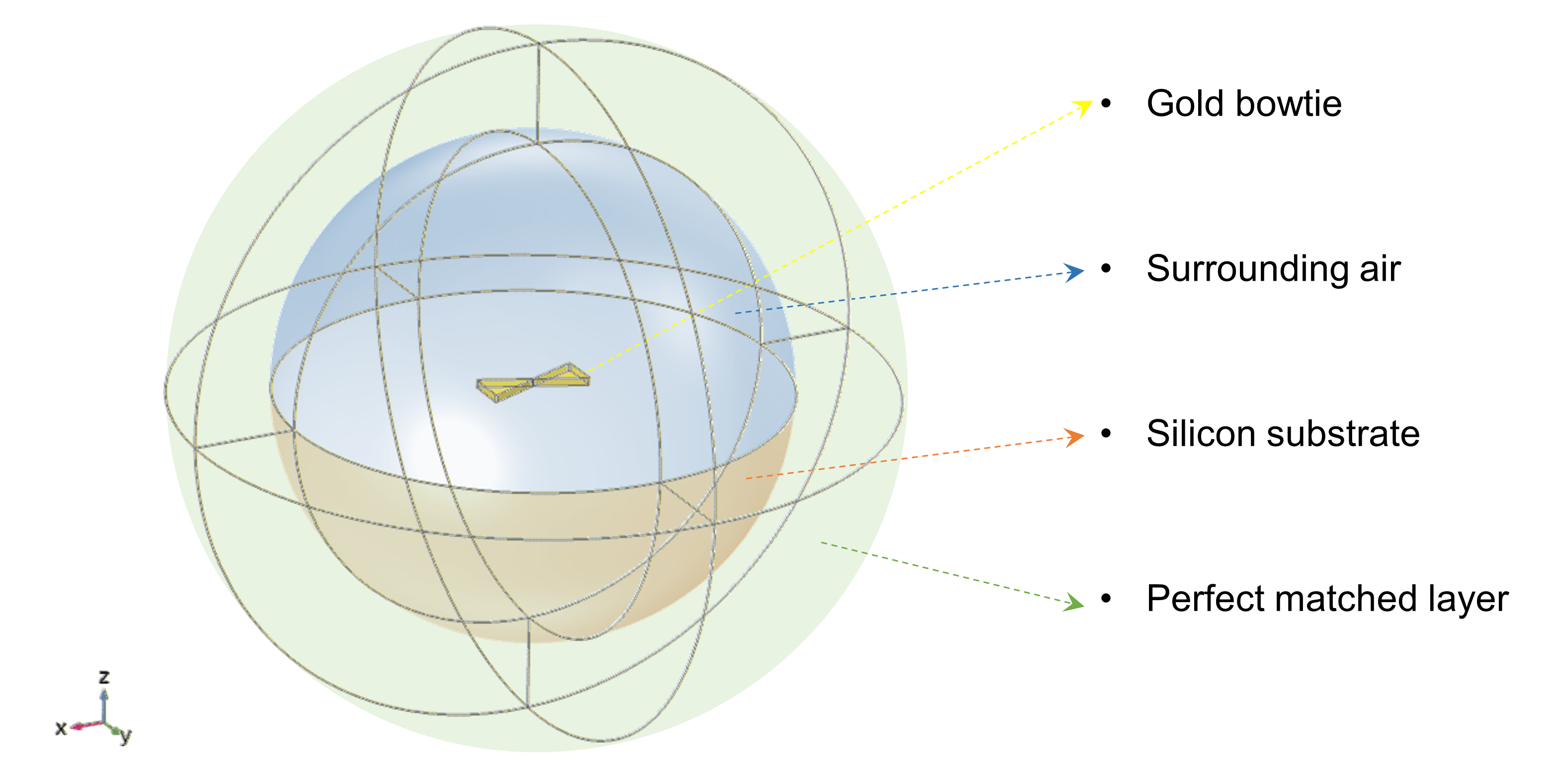
**

**Figure S1.** The overall system in FEM simulation.

**
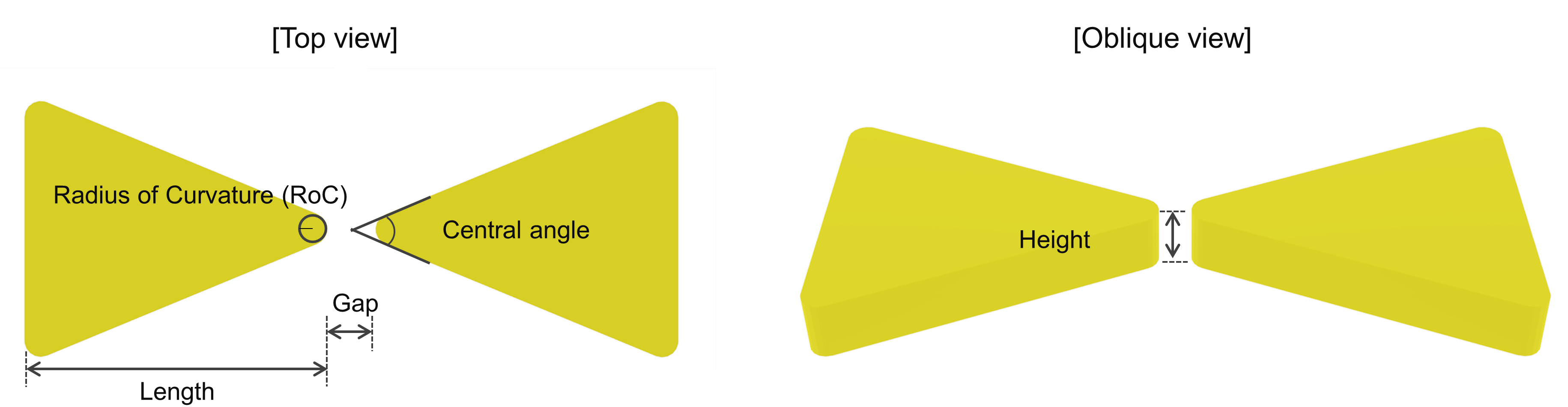
**

**Figure S2.** The geometric parameter sweep to analyze near-field enhancement of Au bowtie nanoantennas.

We swept the geometric parameter in the FEM simulation as follows:

1. central angle – from 20 degrees to 40 degrees. step size: 10 degrees

2. radius of curvature (RoC) – 1, 5, 10 nm.

3. bowtie gap – from 5 nm to 15 nm. step size: 5 nm

**
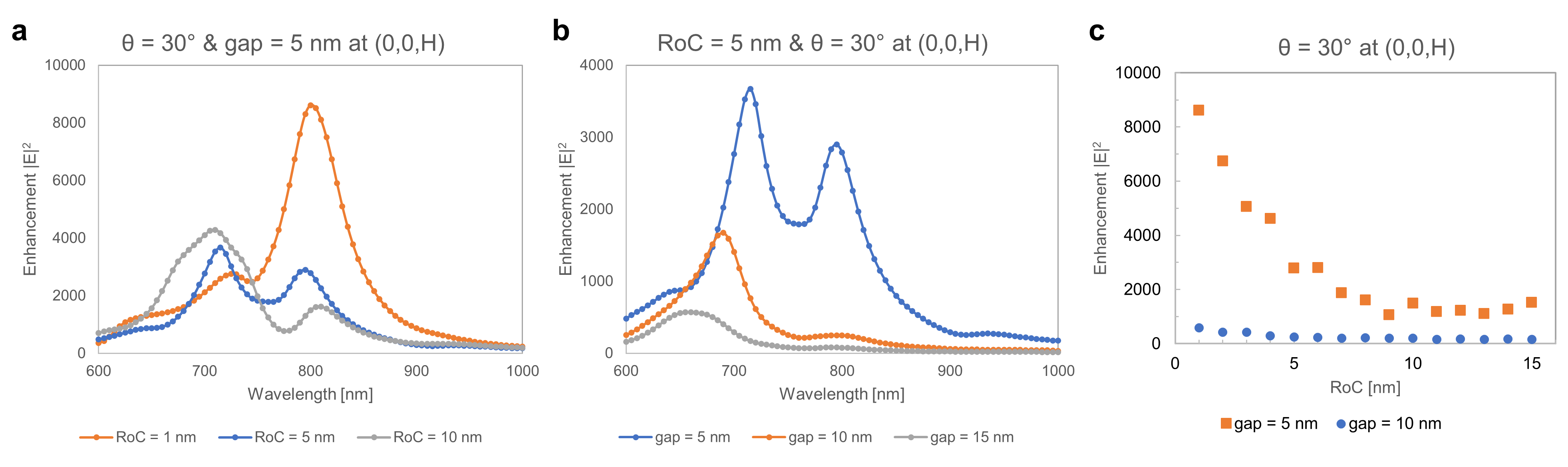
**

**Figure S3.** The results of enhancement |*E*|^2^ from the parametric sweep. (a) The RoC sweep at a fixed central angle of 30˚ and nanogap of 5 nm and (b) nanogap sweep at the fixed central angle of 30˚ and RoC of 5 nm from λ= 600 nm to λ= 1000 nm. (c) Nanogap-dependent RoC sweep at the fixed central angle of 30˚ and λ = 800 nm.


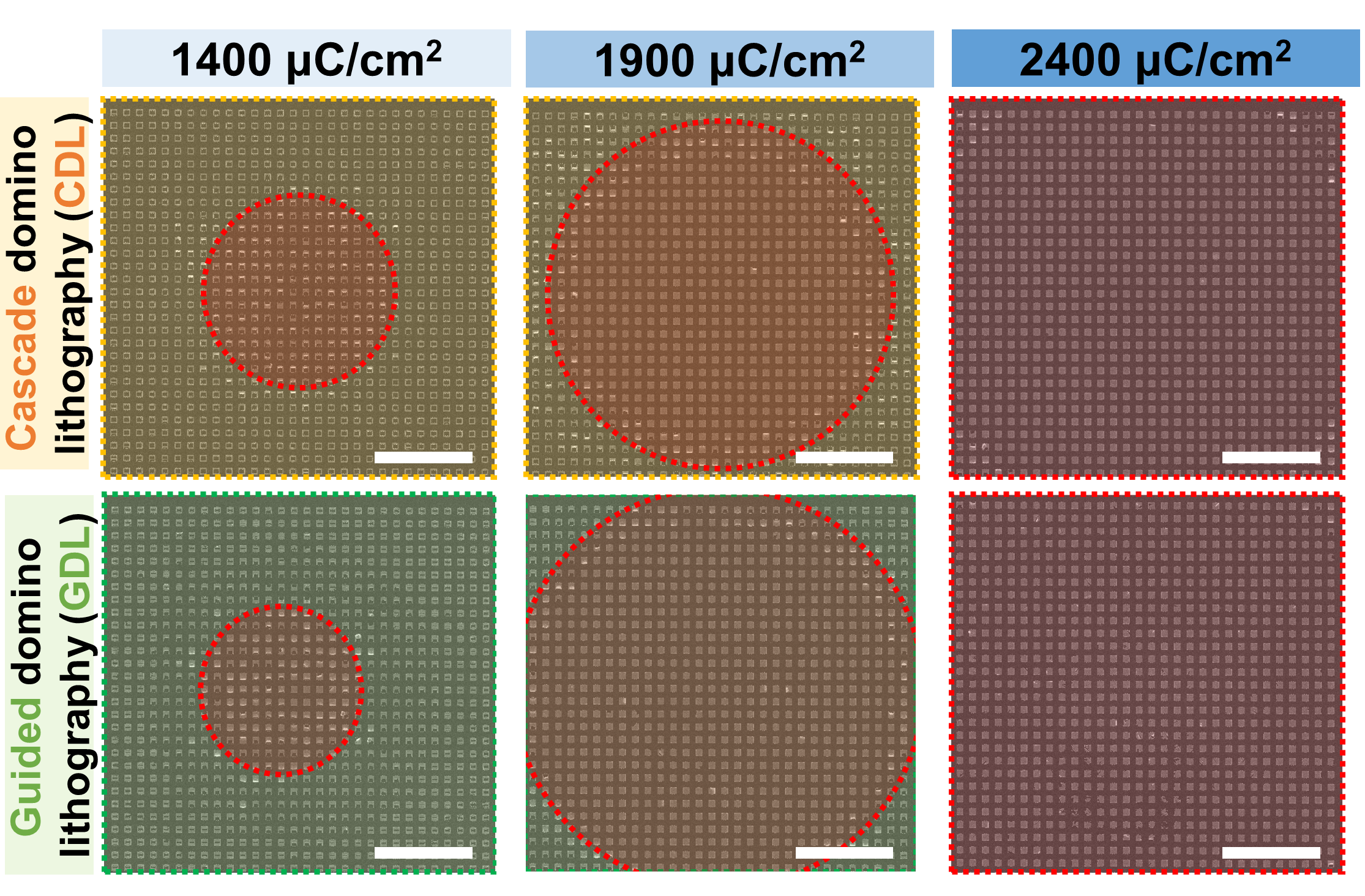


**Figure S4**. SEM images of ultra-sharp bowtie nanoantennas exposed by various exposing doses in CDL and GDL arrays. The development time was 20 min and exposing doses were varied: 1400, 1900, and 2400 µC/cm^2^, respectively. The green area is the region with the uniform fabrication of ultra-sharp bowtie nanoantennas, the yellow area is the region with occasionally misaligned bowtie nanoantennas, and the red area is the region without bowtie nanoantennas due to the absence of collapsed structures by excessive exposing doses. Scale bars: 1 µm.


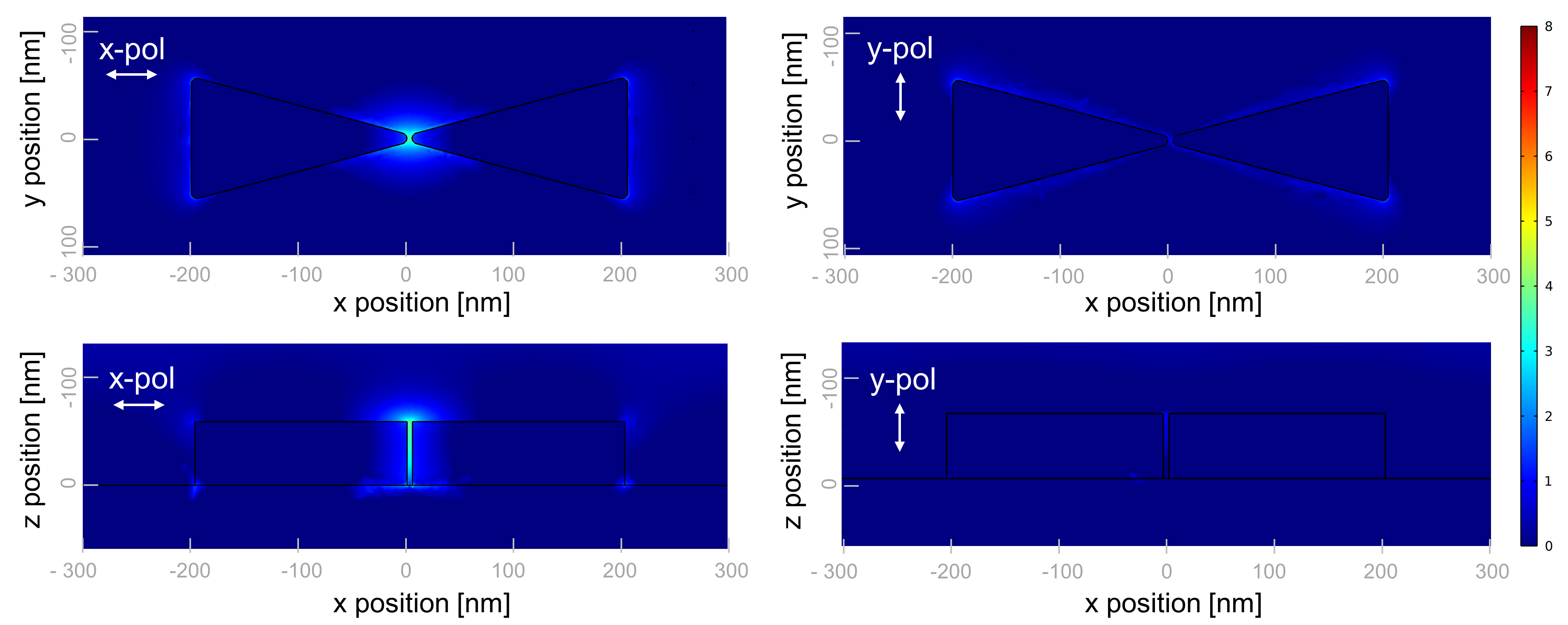


**Figure S5.** The field distributions of $\text{log}_{\text{10}}\text{[}{|E|}^{2}\text{]}$ depending on polarizations at λ = 800 nm.

This figure shows field distributions at λ = 800 under the x-polarization and y-polarization in different planes, respectively. The upper two figures show the distribution on the XY plane (Z = 60 nm) and the bottom two figures show the distribution on the ZX plane (X = 0 nm). In the case of x-polarization, the high field enhancement effect occurs inside the nanogap. Under y-polarization, the most enhanced parts are at the corners of each triangular nanostructure.

**
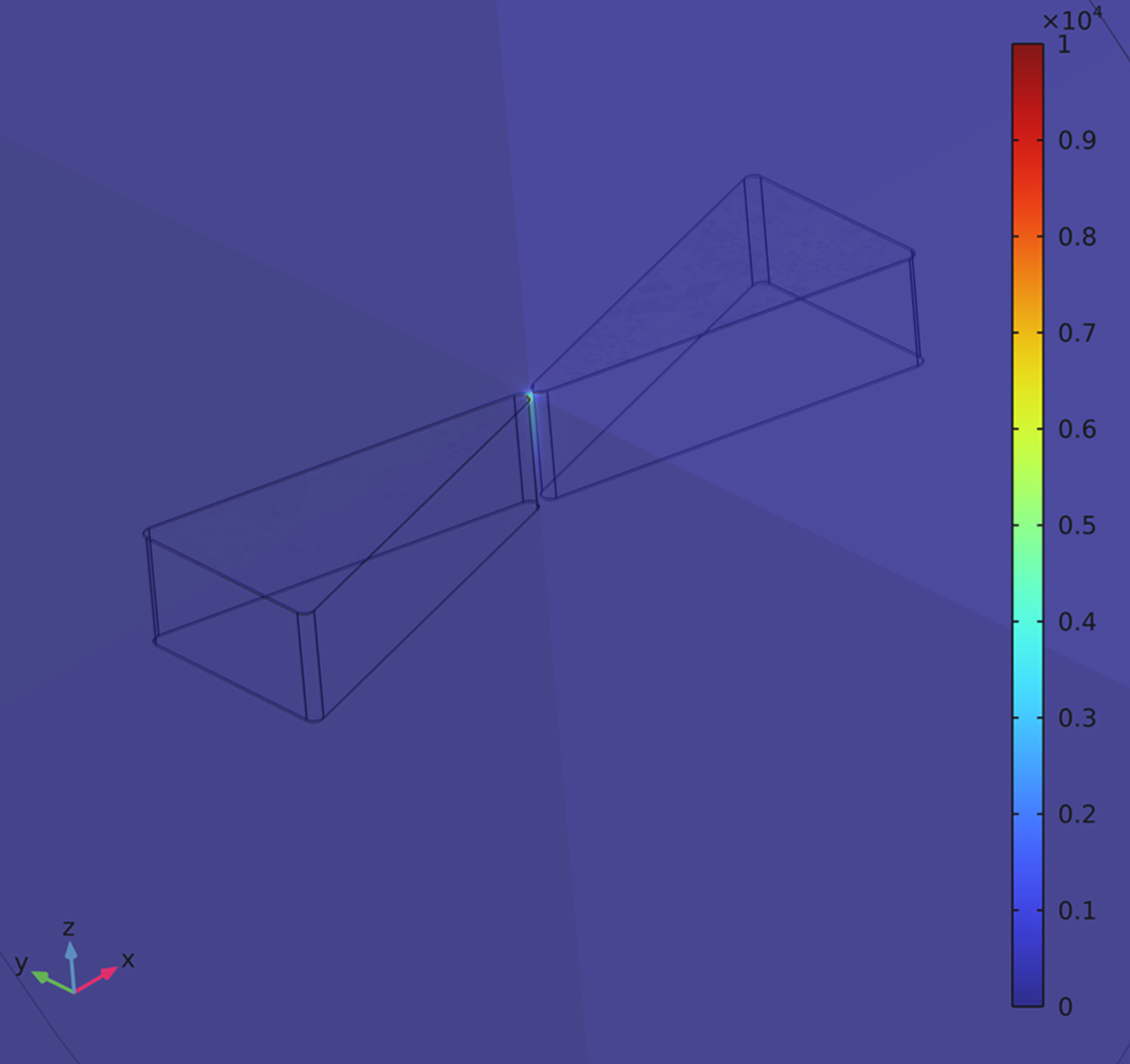
**

**Figure S6.** The three-dimensional distribution of enhancement |*E*|^2^ at λ = 800 nm.
